# Orientation of Blacktip Sharks (*Carcharhinus limbatus*) to Underwater Sound

**DOI:** 10.1093/iob/obag033

**Published:** 2026-06-30

**Authors:** C L Sullivan, E R Gerstein, S M Kajiura

**Affiliations:** Department of Biological Sciences, Florida Atlantic University, 777 Glades Road, Boca Raton, FL 33431, USA; Department of Biological Sciences, Florida Atlantic University, 777 Glades Road, Boca Raton, FL 33431, USA; Department of Biological Sciences, Florida Atlantic University, 777 Glades Road, Boca Raton, FL 33431, USA

## Abstract

Sharks respond to low frequency pulsed sounds but are presumed to lack the capacity to detect these sounds beyond the acoustic near field, where particle motion dominates. This study quantified the distance that blacktip sharks (*Carcharhinus limbatus*) oriented to sound stimuli and determined that responses could be consistently initiated from the acoustic far field. Using an aerial drone, *C. limbatus* were filmed responding to sound stimuli (100–200 Hz; 200–400 Hz; and 400–800 Hz) generated by an underwater speaker. Upon detection, *C. limbatus* elicited a sudden 20–160° turn away from the speaker and rapidly swam away. Sharks responded to all frequencies from at least 62 m, and 71.6% of all responses (*n* = 209) occurred in the far field. Sharks never responded to a high frequency (10 kHz) control stimulus of comparable volume. The sound pressure levels for all stimuli were measured *in situ* and used to model the propagation away from the source. This permitted direct calculation of the stimulus intensity for the point at which each shark initiated a response. A greater sound pressure level was required to elicit a response at higher frequencies, supporting earlier work that demonstrated greatest sensitivity to low frequencies. The ability of blacktip sharks to detect and orient away from a sound stimulus at distances that extend beyond the acoustic near field, suggests that they are detecting particle motion in the far field.

## Introduction

Hearing plays an important function across all vertebrate taxa, and its evolutionary origins began in aquatic animals (Popper and Fay 1993, 1997). The auditory system can potentially enable an animal to determine the location of a sound source, then orient either toward or away from its location (Popper and Hawkins 2018). These two abilities allow an animal to use acoustic stimuli to find prey, avoid predators, locate mates, and detect and react to other important cues (Banner 1972; Hawkins and Popper 2018).

Early studies on shark hearing demonstrated that elasmobranchs are capable of directional hearing, and possibly sound source localization (Popper and Hawkins 2018). Various behavioral and physiological studies provided evidence that elasmobranchs can detect and respond to sounds ranging from 20 to 1500 Hz, with greatest sensitivity between 25 and 200 Hz (Parker 1910; Moulton 1958; Kritzler and Wood 1961; Nelson and Gruber 1963; Nelson and Johnson 1972; Myrburg 2001; Casper and Mann 2009). Elasmobranchs are attracted to overdriven sine waves and filtered broad-band noise (55–1000 Hz), and sound attractiveness increases directly with pulse rate, and inversely with frequency (Nelson and Gruber 1963; Myrberg et al. 1972; Nelson and Johnson 1972).

Several of these studies assessed the behavioral response of free-swimming sharks to a sound stimulus. Nelson and Gruber (1963) successfully attracted Carcharhiniform sharks (*Carcharhinus leucas, Negaprion brevirostris, Galeocerdo cuvier, Sphyrna* sp., and *Carcharhinus* spp.) from great distances using a sound clip that mimicked the low frequency band and pulse rate of a speared grouper. Using a 150–600 Hz noise-band, Myrberg et al. (1978) described a withdrawal response from *C. falciformis* when played at +54 dB/μbar re 1 m, whereas the same stimulus was an attractant when played at +34 dB/μbar. In a subsequent study that examined the response of *N. brevirostris*, Klimley and Myrberg (1979) found that the minimum increase in intensity for these sound stimuli to elicit a withdrawal response was 18 dB above broad-band ambient and concluded that the response of the sharks was determined by signal intensity and the abruptness of signal onset. These studies illustrate that sound stimuli can elicit various reactions (e.g., attraction; avoidance; and startle) from the sharks.

**Fig. 1:**
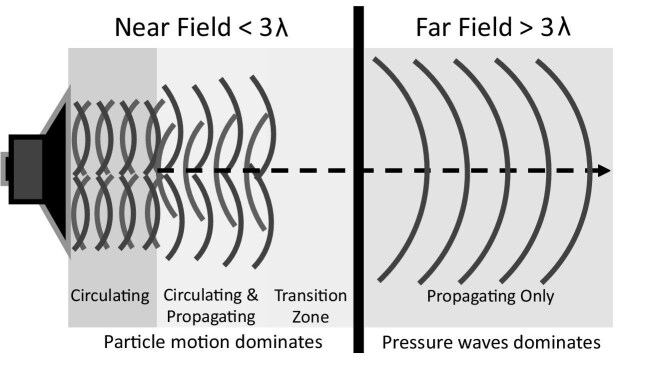
Representation of the acoustic fields. The sound travels from the source in circulating and propagating waves as a hydrodynamic flow dominated by particle motion (near field). As the sound travels beyond three wavelengths from the source, it consists of only propagating waves being dominated by pressure waves (far field). At that distance the sound pressure waves must be transduced, typically by a gas-filled-organ, to stimulate the hair cells of the inner ear to be detected.

When a sound is emitted, it generates a mechanical energy waveform that travels through two acoustic “fields” (Fig. 1; Popper and Fay 1993). Within three wavelengths (<3λ) of the sound source, known as the near field, the sound wave is a complex spherical wave that consists of oscillatory particle motions accompanied by compressions (increase) and rarefactions (decrease) of sound pressure (Popper and Fay 1993; Chapuis and Collin 2022). These near field particle motions (vector quantity) are hydrodynamic flows that attenuate rapidly from the vibrating source and have dominance over sound pressure (scalar quantity; Popper and Fay 1993; Popper and Hawkins 2018). When the sound reaches the far field (>3λ), it occurs as a plane wave (flat waveform), and pressure assumes dominance over particle motion, though particle motion remains present (Popper and Fay 1993; Radford et al. 2012; Nedelec et al. 2016). Under ideal far field conditions (free field), the sound pressure and particle motion are in phase together, and the levels of both parameters decrease 6 dB per distance doubling from the sound source (Hawkins and Popper 2018; Chapuis and Collin 2022).

In the near field, fish utilize their inner ear and lateral line system to directly detect the particle motion component of sound (Higgs and Radford 2013). In the far field, environmental particle motion is still present, and sound pressure detection is mediated by the gas-filled swim bladder, which converts pressure oscillations to particle motion, thus allowing the inner ear to perceive the stimulus (Yan et al. 2000; Casper 2006; Radford et al. 2012). Therefore, it is hypothesized that only fish that possess a swim bladder, in conjunction with the inner ear, can detect the sound pressure component in the acoustic far field. Elasmobranchs do not possess a swim bladder, suggesting that their inner ear detects only the particle motion component of sound (Meredith et al. 2022).

There are two hypothesized mechanisms for elasmobranchs to detect particle motion: *via* the otolithic and non-otolithic pathways. Like all vertebrates, elasmobranchs process sound in the inner ear where the basic anatomy is largely conserved among various elasmobranch species (Sauer et al. 2023a, b; Chapuis et al. 2023). The inner ear consists of three membranous semicircular canals, and three otoconial end organs (saccule, utricle, and lagena), which each house a sensory epithelium (macula; Hawkins and Popper 2018). The semicircular canals allow the animal to detect angular acceleration associated with rotating movements of the body, whereas the maculae directly detect particle motion and are the foundation for directional hearing (Hawkins and Popper 2018; Chapuis and Collin 2022). The macula of each otoconial end organ consists of polarized hair cell bundles sheathed in a mucilaginous cupula overlain with otoconia, which are calcium carbonate crystals that are 3X denser than the fishes’ body (Myrberg 2001; Hawkins and Popper 2018). The dense otoconia experience a greater inertia in response to a generated particle motion, which results in shearing of the hair cells in the direction of the motion (Casper and Mann 2009). This response initiates auditory detection and the potential for the shark to receive directional information about the location of the sound source (Evangelista et al. 2010; Hawkins and Popper 2018).

Elasmobranchs also possess a fourth, non-otoconial end organ, the macula neglecta, that consists of patches of sensory epithelia (one patch in batoids, two patches in sharks) lightly covered by a mucilaginous cupula, similar to the cupula of the lateral line neuromasts (Myrberg 2001; Hawkins and Popper 2018; Chapuis and Collin 2022). Unlike other maculae, the cupula in the macula neglecta is not mass-loaded with otoconia (Meredith et al. 2022). It is hypothesized that acoustic pressure above the head is concentrated on the fenestrae ovali at the base of the parietal fossa on the neurocranium (Corwin 1989). The particle displacement caused by the acoustic pressure induces fluid flow within the posterior canal that contains the macula neglecta (Meredith et al. 2022). The fluid flow causes movement of the mucilaginous cupula that shears the hair cells of the macula neglecta resulting in sound detection via a non-otolithic pathway (Casper and Mann 2009).

The macula neglecta is sensitive to vibrations and gravitational changes (Lowenstein and Roberts 1951), properties that may be critical for a shark’s sound perception (Tester et al. 1972). Corwin (1983) found that the vibrational sensitivity of the macula neglecta increases directly with hair cell numbers; in sharks, these increase proportionally with age and body size (Casper et al. 2003). The number of sensory hair cells in the macula neglecta has been quantified for 20 elasmobranch species (Corwin 1978, 1981, 1983; Barber et al. 1985; Casper et al. 2003; Sauer et al. 2022a, b, 2023b). In two Carcharhinid species, numbers range between 200,000 (*C. melanopterus*) to 260,000 (*C. falciformis*; Corwin 1978, 1981). The latter species was found to have the largest macula neglecta and the greatest number of hair cells of any vertebrate (Corwin 1978). In a more recent study, Sauer et al. (2023b) documented a significantly larger macula neglecta and a greater number of hair cells in *Carcharhinus brachyurus* compared to the other 8 non-carcharhinid species in the study. These studies suggest that the large macula neglecta of carcharhinid sharks may result in more specialized hearing capabilities compared to other species and may explain why these non-demersal sharks seem to possess the greatest sensitivity to sound stimuli (Corwin 1978; Casper et al. 2003; Sauer et al. 2023b).

Despite the various studies that have examined the hearing capabilities of elasmobranchs, none have quantified shark hearing nor sound source localization in the acoustic far field. This is because there was no method available to simultaneously measure both the shark’s behavioral response and the distance of the shark from that sound source when the shark was in the acoustic far field. Recent advances in aerial drone technology now allow remote viewing and the potential to quantify orientation to far field stimuli. Therefore, the purpose of this study was to (1) quantify the distance at which free-swimming sharks responded to low frequency pulsed sound stimuli located in the far field, and (2) determine whether a shark could localize the sound source.

## Methods

### Study animal

The animal chosen for this study was the blacktip shark, *Carcharhinus limbatus* (Müller and Henle 1839). This species is found in warm temperate to tropical waters around the world (Castro 2011). Each winter in the Western Atlantic (January–March), blacktip sharks form large aggregations off the coast of Palm Beach, FL, USA (Kajiura and Tellman 2016); smaller populations of residents remain year-round. As a meso-trophic level predator, *C. limbatus* preys on teleosts, cephalopods, crustaceans, and other small elasmobranchs (Castro 2011) and is prey to larger elasmobranchs such as the great hammerhead shark, *Sphyrna mokarran* (Doan and Kajiura 2020). The predictable increased abundance of this species in the clear shallow water off the coast make it an ideal subject to aerially visualize and observe natural behavior when presented with an acoustic stimulus.

### Study site

**Fig. 2:**
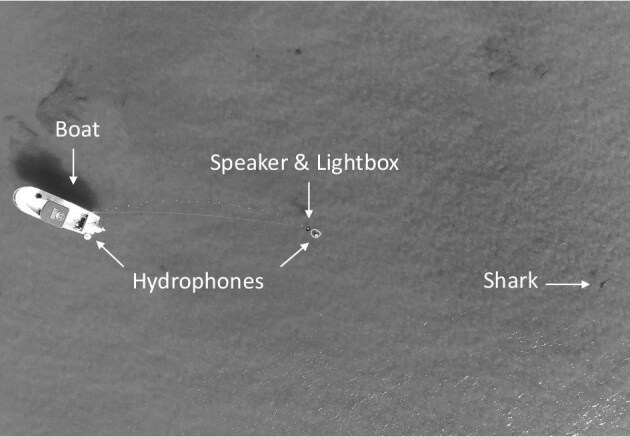
Spatial representation of the experimental set up. The boat is anchored in 2–5 m depth of water with the underwater speaker suspended 1 m below the surface and located ∼13–19 m from the boat. The known length of the boat calibrates the scale to measure the distance at which the shark responds to the sound stimulus.

This study took place off the coast of Southeast Florida near the Jupiter Beach inlet, the Palm Beach inlet, or the Pompano Beach inlet, in areas known to have large aggregations of blacktip sharks. Experiments were conducted from an anchored boat (2–5 m depth) using an underwater speaker to project sound stimuli, in conjunction with 2 recording hydrophones (Fig. 2).

### Acoustic stimuli

**Fig. 3:**
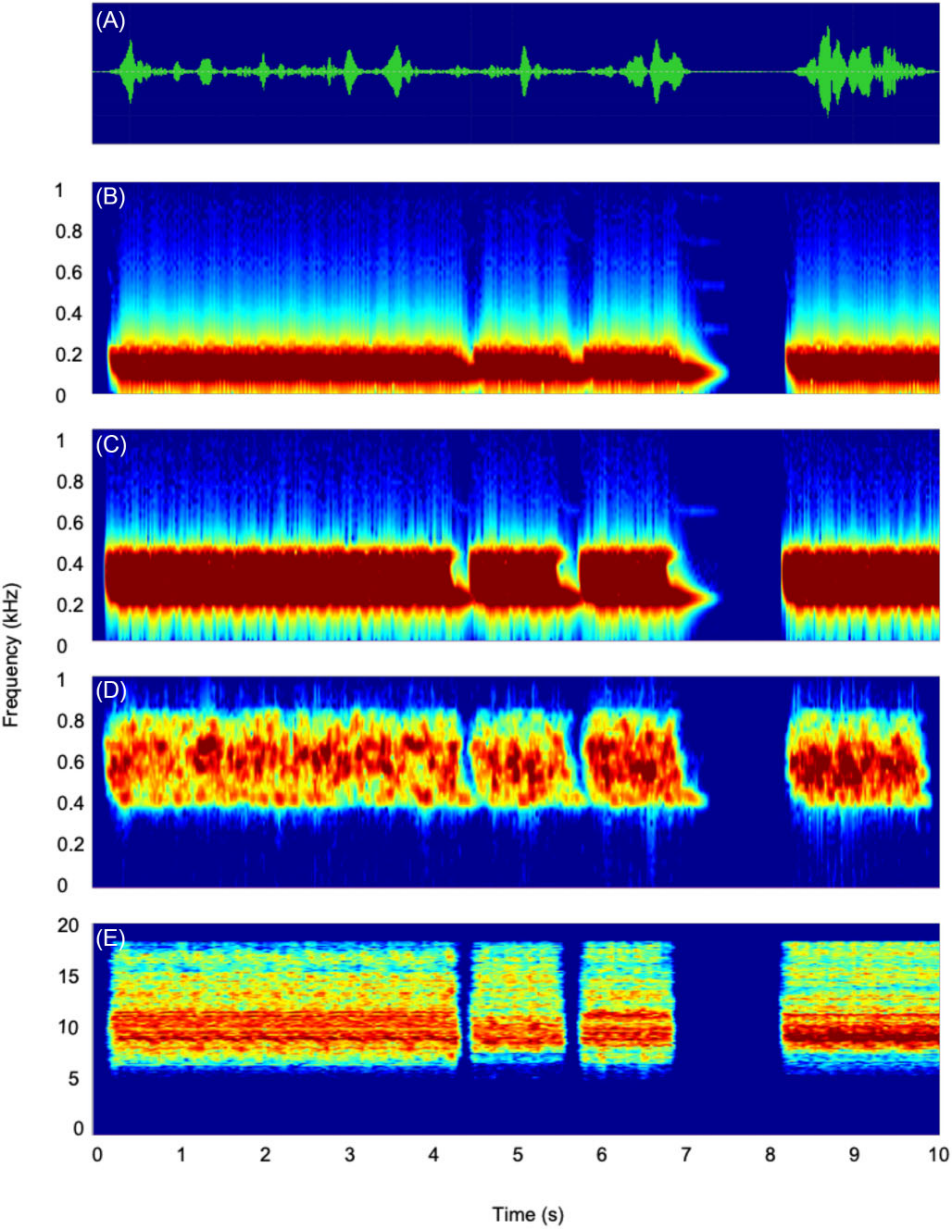
Stimulus waveform (A) and spectrograms of the three experimental stimuli (B: 100–200 Hz; C: 200–400 Hz; and D: 400–800 Hz), and the control stimulus (E: 10,000 Hz). The spectrograms depict the intensity (red = high intensity, blue = low intensity; dB; *z*-axis) of frequency (kHz; *y*-axis) vs. time (s; *x*-axis).

We used three experimental acoustic stimuli and one control in this study. The base waveform consisted of amplitude modulated, intermittent pulse trains of filtered random noise (25–100 Hz, 15–7.5 decreasing pulses s^−1^) (modified from Nelson and Johnson 1972) (Fig. 3A). The three experimental stimuli consisted of irregularly pulsed frequencies of 100–200 Hz, 200–400 Hz, and 400–800 Hz. The stimuli were created by using a high and low pass band filter to ensure that only the desired frequencies were transmitted. The band pass filter was 99–201 Hz for the 100–200 Hz sound, 199–401 Hz for the 200–400 Hz sound, and 399–801 Hz for the 400–800 Hz sound (Fig. 3B–D). This method preserved the temporal pattern and amplitude fluctuation of the original stimulus, and pitch shifted the stimulus to higher frequency ranges. The control stimulus was a 10 kHz, irregularly pulsed frequency well established to be outside of the elasmobranch hearing range (Fig. 3E). The output volume was kept the same for each stimulus throughout the study.

Stimuli were presented from an Apple MacBook Pro computer and amplified through a Sony^®^ XM-1252GTR Stereo Power amplifier powered by a 12-volt marine deep cycle battery. The output from the amplifier was transmitted to a Clark Synthesis AQ339 Diluvio™ Underwater Loudspeaker (frequency range of 20 Hz–17 kHz). The speaker was suspended 1 m below the ocean surface.

To record the sound pressures generated by the AQ339 speaker, a USRD F37 hydrophone (frequency range 10 Hz–37 kHz with a sensitivity of −201 dB) was suspended at a depth of 1 m below the surface, at a distance of 1 m from the speaker. Another USRD F37 hydrophone was suspended at a depth of 1 m below the surface off the side of the boat, situated approximately 13–19 m from the speaker. The two hydrophones continuously recorded to individual M-audio Microtrack 24/96 2-channel digital recorders, with a sampling rate of 44.1 kHz. The data collected from these hydrophones were used to determine the sound pressure level (SPL) and frequency range of each stimulus underwater.

### Field measurements

To establish how the pulsed stimuli were propagated from the speaker, the sound field at the testing site was mapped. The boat was anchored in the testing area and the speaker was anchored 13–19 m from the boat and suspended 1 m below the surface. The distance and depth of the speaker were representative of the actual deployment distance and depth during experimental trials and the speaker faced the same direction for the duration of the measurements.

**Fig. 4:**
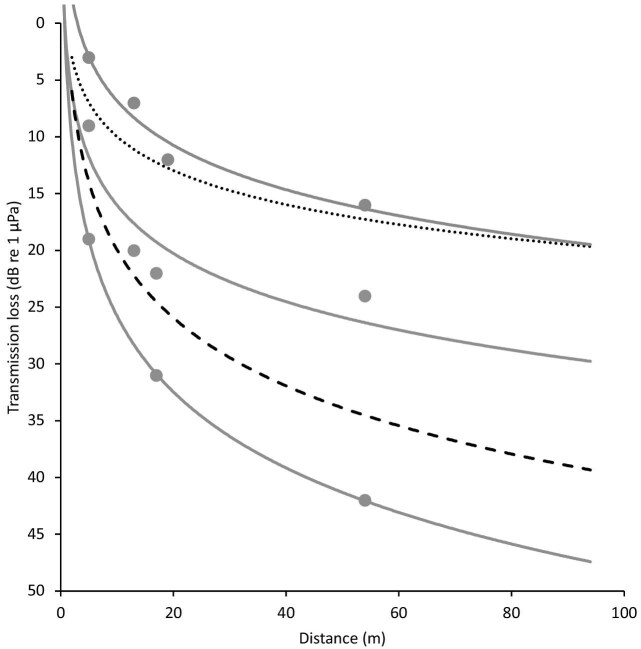
Transmission loss of the peak frequencies of projected sound clips at the study site. Curves are fit for the measured values for the 120 Hz peak of the 100–200 Hz clip (red), the 250 Hz peak of the 200–400 Hz clip (green), and the 500 Hz peak of the 400–800 Hz clip (blue). The black dotted line is propagation assuming cylindrical spreading, and the dashed line is propagation assuming spherical spreading. Experiments were conducted in a shallow area with a sandy substrate, causing greater transmission loss for lower frequencies.

Four calibrated hydrophones, each suspended 1 m below the surface, were radially anchored around the speaker, and each recorded to an M-audio Microtrack 24/96 2-channel digital audio recorder sampling at 44.1 kHz. The control stimulus and three experimental stimuli were sequentially presented from the speaker for 10 s each and at the same volume. Trials were repeated with the four hydrophones located at 1, ∼10, ∼20, ∼30, and ∼60 m from the speaker. The recordings were analyzed using a Model SR770 FFT Network Analyzer using a Blackman–Harris window. The 4 sound clips (control and 3 experimental stimuli) recorded by each radially spaced hydrophone at each distance were individually played through the network analyzer. For each stimulus, the network analyzer took an RMS averaging, which yielded the average dB level per frequency. The root mean square (RMS) peak values recorded at the 1 m hydrophone served as source level values, which were measured at 140 dB for each of the experimental frequencies (100–200 Hz, 200–400 Hz, 400–800 Hz, and 10 kHz). The difference in greatest peak value at each radial distance (∼10, ∼20, ∼30, and ∼60 m) was subtracted from the 1 m source level to generate a propagation transmission loss function for the peak frequencies of each stimulus at the study site (Fig. 4).

**Fig. 5:**
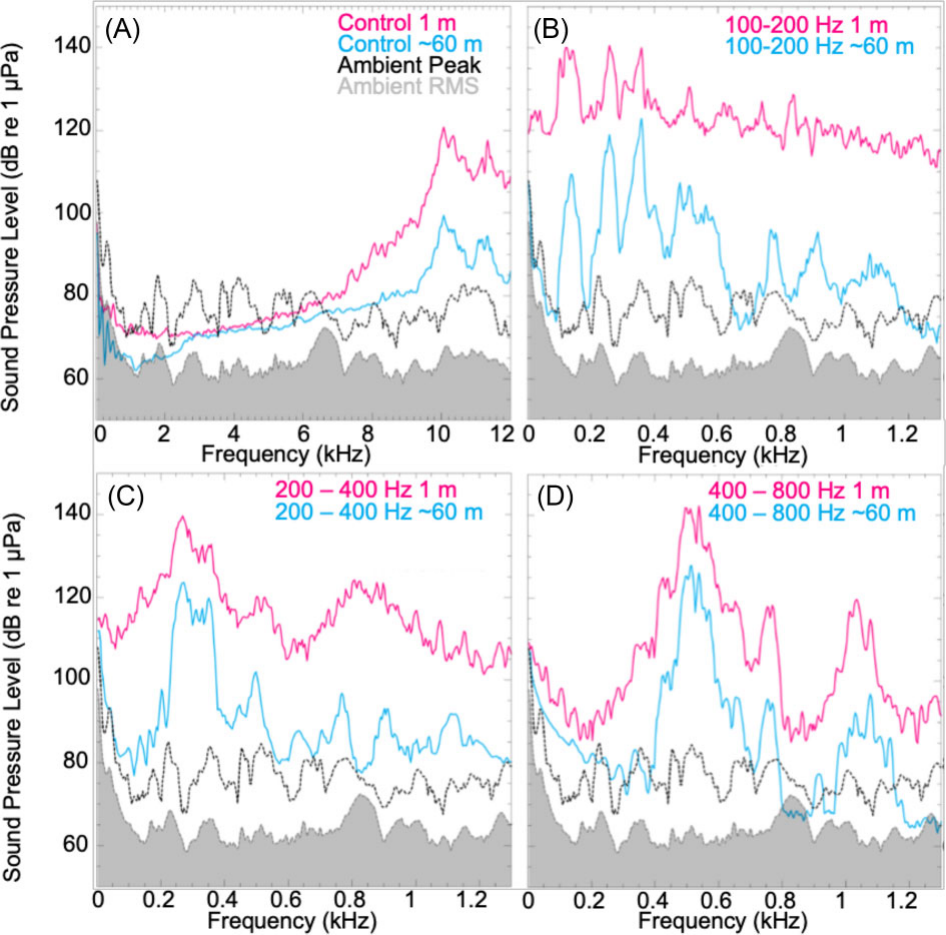
Stimuli spectrums. Spectrums show the SPL vs. frequency of each stimulus when played 1 m (pink line) and ∼60 m (blue line) from the hydrophone. The RMS (gray area) and peak hold (black dotted line) of the lowest observed ambient noise levels are compared to the SPLs of each stimuli. (A) The RMS Spectrum of the 10,000 Hz pulsed control sound clip. (B) Peak hold spectrum of the 100–200 Hz pulsed stimulus. (C) Peak hold spectrum of the 200–400 Hz pulsed stimulus. (D) Peak hold spectrum of the 400–800 Hz pulsed stimulus.

In addition to mapping the sound propagation at the study site, the acquired data were used to create spectra graphs for each stimulus that showed the relationship between the SPL and frequency (Fig. 5). To make a spectra graph, the stimulus recorded at 1 m (source level) and at ∼60 m (the farthest distance we measured) was played back through the network analyzer (same window as previously described). Using the peak hold averaging setting (600 spectra per signal), the peak spectral magnitudes of the stimulus were detected and displayed. RMS and peak hold dB values were also generated for ambient noise using a portion of the recording where observed intensity levels were the lowest. All dB values were then plotted, comparing the SPLs of the stimulus (1 m and at ∼60 m) to the ambient noise.

### Experimental protocol

Experiments were conducted between 7:30 am and 12:30 pm on days with minimal wind to reduce wave-generated surface distortion and associated noise. To begin an experimental trial, a DJI Phantom 4 Pro aerial drone was launched from the boat and flown in the area to locate a shark aggregation. The boat was anchored in nearshore waters (2–5 m depth) near the sharks and the engine was turned off. The Clark Synthesis AQ339 Diluvio™ Underwater Loudspeaker was deployed from the boat and drifted with the current to approximately 13–19 m off the stern of the boat. This distance minimized the effect of the presence of the boat on the shark’s responses. A remotely controlled lightbox floated above the speaker (Fig. 2). When a sound was played, the skyward facing lightbox was activated to provide a visual indicator to the drone. The aerial drone hovered at an altitude of ∼40–50 m above the vessel. The drone camera was oriented directly downward to include the boat, lightbox, and speaker, as well as the surrounding water. At 40 m altitude the field of view was 42.1 m × 74.8 m; at 50 m altitude the field of view was 52.8 m × 93.5 m.

When a shark swam into frame, it was subjected to 5 s of the control stimulus (10 kHz irregularly pulsed sound), a 5 s period of ambient noise (no sound stimulus), then one of the three randomly chosen experimental stimuli. The sound playback level was 140 dB re 1 µPa re 1 m which yielded a source level of approximately 80 dB above the lowest documented ambient values. The stimulus presentation continued until the shark responded or swam out of frame. All experiments were conducted under FAU IACUC protocol A20-05.

### Quantifying acoustic detection

**Fig. 6:**
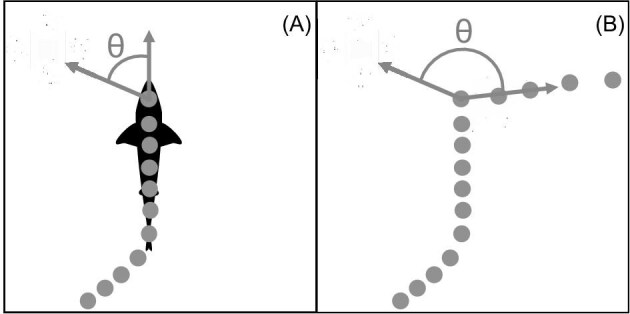
Turning response of a shark when presented with an acoustic stimulus. (A) The orientation of the shark with respect to the speaker prior to the onset of the acoustic stimulus, and (B) after it turns away from the speaker upon sound production. The blue dots show the position of the shark’s snout in each frame of video prior to and after stimulus presentation. The pink arrows show the angle ($\theta$) measured.

The video footage captured by the aerial drone was analyzed to quantify orientation distance and behavior. Sharks were deemed to have detected the sound stimulus if they initiated a sudden change in trajectory immediately following the onset of the stimulus. Data were omitted during trials in which stimulus onset coincided with the presence of other variables (i.e., colliding sharks, fish, boat wake, etc.), or when the behavioral response was ambiguous. To quantify the change in trajectory a dot was placed in the center of the sharks’ head for each frame of video using the software package Logger Pro 3.16.2. The resulting sequence of points depicted the trajectory of the shark, before and after sound production. This image was analyzed using the Angle tool in the software package ImageJ 1.53a. The angle of the initial trajectory, prior to turning in response to the sound stimulus, was measured with respect to the speaker (Fig. 6A). The angle of the trajectory after turning was also measured with respect to the speaker, and with respect to the initial trajectory (Fig. 6B).

To quantify the distance at which the shark initiated its response, the video frame of the shark immediately preceding its turn was imported into the analysis software ImageJ 1.53a and the known length of the boat (6.73 m), was used to calibrate the scale. The Straight-Line tool in ImageJ was used to measure from the speaker to the tip of the sharks’ snout and yielded a calibrated distance in meters. The distances that the near field extended (3λ) were calculated for the lowest frequency for each stimulus (i.e., 100, 200, and 400 Hz). Far field detection was assumed if the shark responded at a distance exceeding that calculation (Table 1). From the measured distances and the calculated propagation functions for each frequency, we derived the SPL (dB re peak ambient level) for each orientation.

**Table 1:** The calculated wavelength and distance at which the far field begins for each sound stimulus tested.

Frequency (Hz)	λ (m)	Far field (3λ) (m)
100–200	15	≥45
200–400	7.5	≥22.5
400–800	3.75	≥11.3
10,000 (Control)	0.15	≥0.5

*Note:* For each stimulus, the lowest frequency in the range was used to calculate the far field distance. This yielded the most conservative estimate of far field distance.

### Analysis

We tested whether the distribution of trajectories differed pre- and post-sound production for each frequency using a Watson–Williams test, which is specific for testing angular data (Zar 1999). For each response the difference in pre- and post-stimulus trajectories was log-transformed to achieve normality and homoscedasticity and an analysis of variance (ANOVA) was applied to determine if they differed among the three experimental frequencies.

The mean distance at which a response was initiated, or at which the stimulus was presented but no response was initiated, was compared within and among the three frequencies using an ANOVA on the log-transformed data. If differences were found, a Scheffe post-hoc test was applied to determine which pairs differed.

An ANOVA was used to test whether the calculated SPL (re peak ambient level) for all orientations differed among the three frequencies and a Scheffe post-hoc test was applied to determine which pairs differed. The data were log transformed to achieve normality prior to testing.

## Results

### Acoustic stimulus

We measured the sound characteristics and propagation around the speaker for the three experimental stimuli and the control. The peak frequencies for each of the 3 experimental stimuli were 120 Hz for 100–200 Hz, 250 Hz for 200–400 Hz, and 500 Hz for 400–800 Hz. The transmission loss curve was calculated for the peak frequencies of each of the stimuli: 120 Hz, *y* = 4.1348ln(*x*)–1.5858, *R*^2^ = 0.999; 250 Hz, *y* = 6.6008ln(*x*) + 0.4827, *R*^2^ = 0.897; 500 Hz, *y* = 10.509ln(*x*) + 0.8482, *R*^2^ = 0.978 (Fig. 4). As the sound propagated, each signal appeared to lose the greatest amount of its source level energy within approximately 10 m from the sound source. The decay rate was inversely proportional to frequency, with greater transmission loss at low frequencies and less transmission loss at the higher frequency. Stimuli at 120 and 250 Hz appeared to exhibit a more dramatic decay than the 500 Hz stimulus (Fig. 4). The propagation was uniform in each direction from the speaker. The hydrophones recording at the same distances (i.e., 2 hydrophones located 10m on either side of the speaker) experienced similar values, only differing by 1–2 dB. Therefore, the propagation was primarily frequency dependent, with the depth at which the speaker was projecting being the limiting factor for frequency attenuation. The topography from the shallowest to the deepest part of the site differed by less than 2 m. Any differences in depth provided only a negligible effect on stimulus transmission loss.

### Response orientation

Sharks were exposed to both a control (10 kHz) and one of three experimental frequencies (100–200 Hz, 200–400 Hz, and 400–800 Hz). A positive response to the stimulus was characterized by the shark’s immediate change in trajectory, accompanied by a rapid swim away from the area (Movie 1). Alternatively, a lack of deviance from the shark’s initial behavior was regarded as no response. Sharks never exhibited a response to the control stimulus (*n* = 209), but did exhibit a response to all three experimental treatments. Sharks responded 81.9% (59 out of 72 presentations) of the time to the 100–200 Hz stimulus, 87.0% (47 out of 54 presentations) of the time to the 200–400 Hz stimulus, and 71.1% (59 out of 83 presentations) of the time to the 400–800 Hz stimulus.

**Fig. 7:**
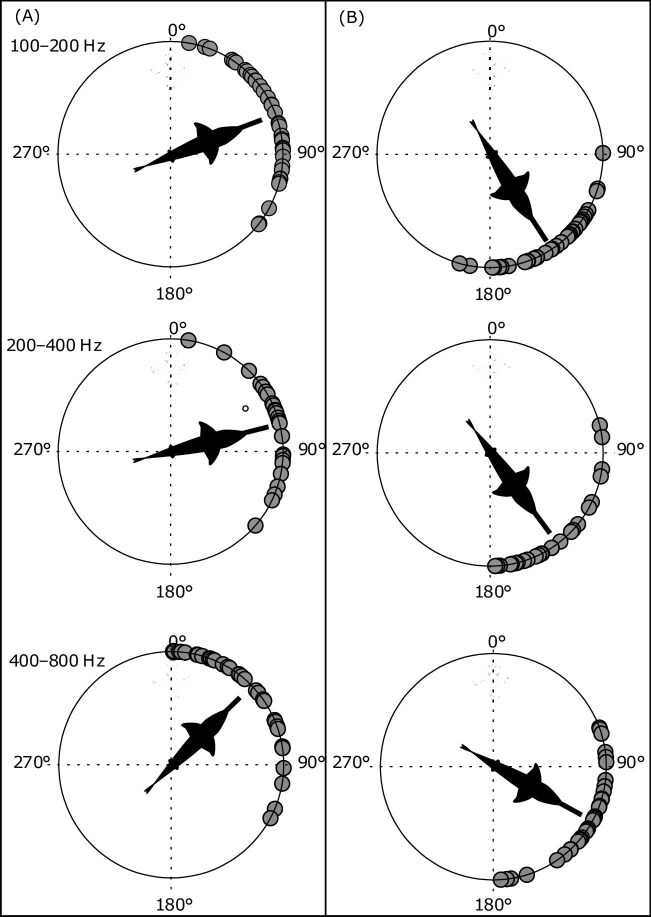
Trajectory of the sharks (A) before and (B) after presentation of an acoustic stimulus. 0^o^ represents the position of the speaker, and the shark indicates mean angle. Sharks exhibited a significant change in trajectory and orientation away from the speaker after sound production. Sharks’ orientation from the speaker, prior to sound production, was the same among stimuli (A; red: 100–200 Hz, green: 200–400 Hz, and blue: 400–800 Hz). Similarly, there was no difference among the stimuli when comparing the sharks’ orientation from the speaker after sound detection (B).

Shark trajectory angle with respect to the speaker was determined for all three frequencies prior to the onset of a treatment stimulus. Sharks swam at a mean trajectory angle of 69.5° ± 30.58° s.d. (range 9.5–128.4°, *n* = 38) for the 100–200 Hz stimulus, 75.7° ± 27.25° s.d. (range 9.1–131.0°, *n* = 26) for the 200–400 Hz stimulus, and 46.6° ± 32.69° s.d. (range 1.2–118.1°, *n* = 33) for the 400–800 Hz stimulus (Fig. 7A). Upon sound production, sharks changed their mean trajectory angle to 146.9° ± 24.38° s.d. (range 89.3–195.6°) for the 100–200 Hz stimulus, 142.3° ± 28.78° s.d. (range 75.9–177.4°) for the 200–400 Hz stimulus, and 118.9° ± 28.03° s.d. (range 69.8–176.2°) for the 400–800 Hz stimulus (Fig. 7B). The mean post-stimulus trajectory differed from the mean pre-stimulus trajectory for all three frequencies: 100–200 Hz (Watson–William’s, *F* = 139.4, *P* < 0.001), 200–400 Hz (Watson–William’s, *F* = 74.3, *P* < 0.001), and 400–800 Hz (Watson–William’s, *F* = 91.7, *P* < 0.001). The mean change in trajectory was 77.3° ± 22.42° s.d. for the 100–200 Hz stimulus, 66.6° ± 22.19° s.d. for the 200–400 Hz stimulus, and 72.2° ± 25.04° s.d. for the 400–800 Hz stimulus. The mean change in trajectory did not differ among the three frequencies (ANOVA, *F* = 2.060, *P* = 0.133).

### Response distance

**Fig. 8:**
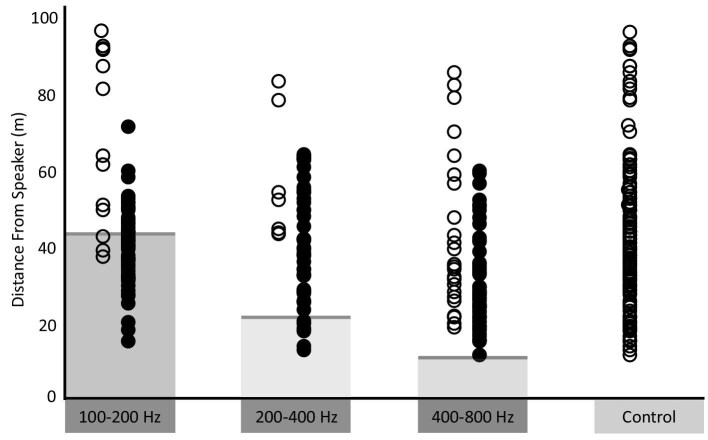
The distance of the shark from the sound source during sound production for each frequency range. Solid circles represent a turn response and open circles indicate a lack of response. The colored bars (red: 100–200 Hz, green: 200–400 Hz, and blue: 400–800 Hz), denote the end of the near field (shaded), indicating most responses occurred in the acoustic far field. There were no responses to the control stimulus (gray: 10,000 Hz).

The mean distance at which a response was initiated, or the stimulus was presented but no response was initiated, was compared for each of the three frequencies. The mean response distances were 40.7 m ± 10.69 m s.d. (range 15.0–74.0 m, *n* = 59) for the 100–200 Hz stimulus, 41.0 m ± 15.56 m s.d. (range 12.4–66.1 m, *n* = 47) for the 200–400 Hz stimulus, and 30.0 m ± 12.81 m s.d. (range 11.1–61.8 m, *n* = 59) for the 400–800 Hz stimulus (Fig. 8). The mean distance of response differed among the three frequencies (ANOVA, *F* = 15.014, *P* < 0.001). The mean distance of response for the 400–800 Hz stimulus was less than the distance for the 100–200 Hz and 200–400 Hz stimuli (Scheffe; *P* < 0.001 for both). There was no difference in the mean distance of response between the 100–200 Hz and 200–400 Hz stimuli (Scheffe; *P* = 0.887).

The mean distance at which a stimulus was presented but no-response was observed was 70.4 m ± 23.81 s.d. (range 38.0–99.9 m, *n* = 13) for the 100–200 Hz stimulus, 58.9 m ± 17.55 s.d. (range 44.3–86.3 m) for the 200–400 Hz stimulus, and 43.9 m ± 21.56 s.d. (18.9–88.8 m) for the 400–800 Hz stimulus (Fig. 8). The mean distance at which the stimulus was presented but no response was observed differed among the three frequencies (ANOVA, *F* = 7.153, *P* = 0.002). The mean distance of no response was greater for the 100–200 Hz stimulus compared to the 400–800 Hz stimulus (Scheffe; *P* = 0.003). The mean distance of no-response for the 200–400 Hz stimulus did not differ from the 100–200 Hz (Scheffe; *P* = 0.733) or the 400–800 Hz (Scheffe; *P* = 0.136) stimuli. Responses were initiated from a closer distance than no responses for all three frequencies (ANOVA, *F* = 32.675, *P* < 0.001 for the 100–200 Hz stimulus; *F* = 562.787, *P* < 0.001 for the 200–400 Hz stimulus; *F* = 11.667, *P* = 0.001 for the 400–800 Hz stimulus).

The majority (71.5%) of the 165 responses to the sound stimuli occurred from the acoustic far field. In total, 33.9% of responses to the 100–200 Hz stimulus, 87.2% of responses to the 200–400 Hz stimulus, and 96.6% of responses to the 400–800 Hz stimulus occurred in the far field. Out of a total of 45 trials in which no-response was recorded, only 3 no-responses occurred in the near field.

**Fig. 9:**
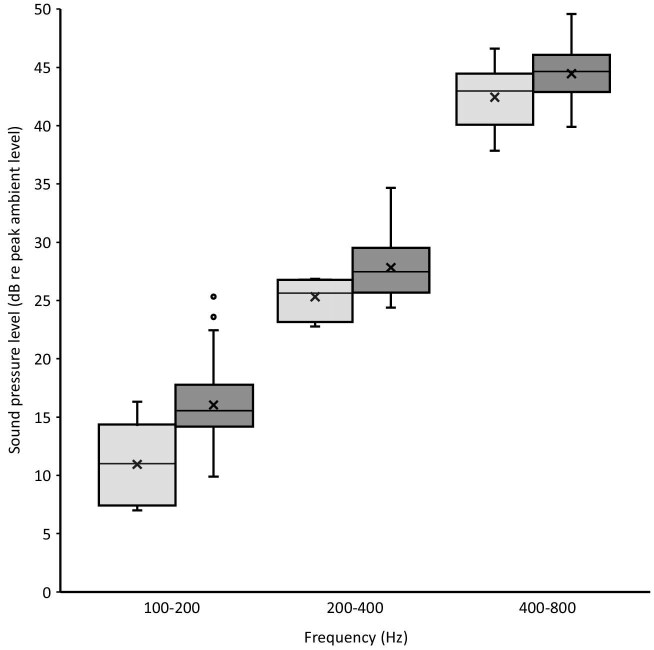
SPL that elicited a response to each of the frequencies. The sound pressure level required to elicit a response increased with frequency. The 100–200 Hz stimulus required a SPL of only ∼16 dB above ambient to elicit a response whereas the 400–800 Hz stimulus required a SPL of ∼45 dB. For each frequency, the SPL that elicited a response (darker boxes) was greater than the SPL that failed to elicit a response (lighter boxes).

The mean SPL that resulted in the initiation of a response was 16.0 dB for the 100–200 Hz stimulus, 27.8 dB for the 200–400 Hz stimulus, and 44.6 dB for the 400–800 Hz stimulus (Fig. 9). The SPL that elicited a response differed among the three frequencies (ANOVA, *F* = 247.17, *P* < 0.001) and post-hoc tests revealed significant differences between each of the frequencies (Scheffe, *P* < 0.001 for all pairwise comparisons). For each frequency, the SPL that elicited a response was greater than the SPL that failed to elicit response (Scheffe, *P* < 0.001 for all pairwise comparisons).

## Discussion

This study quantified the response of free-swimming sharks in the wild when they were exposed to pulsed low frequency sound. This was accomplished by using an aerial drone to unobtrusively film the response of blacktip sharks in the nearshore environment. We found that the sharks exhibited a dramatic turn away from the speaker at all frequency ranges and at distances that extended into the acoustic far field.

### Acoustic stimulus

Preliminary trials, which were not included in any statistical analysis, were conducted using the sound stimulus created by Nelson and Johnson (1972) to establish the model of behavioral response to an acoustic stimulus. The stimulus was a low frequency band (25–100 Hz) created from a random-noise generator, and intermittently pulsed (15–7.5 decreasing pulses s^−1^) by a tone-burst generator (Nelson and Johnson 1972). This audio clip has been demonstrated to be attractive to sharks, when presented at an intensity less than 38 dB above ambient levels (Nelson and Johnson 1972). However, sharks display avoidance behavior when presented with an abrupt sound intensity increase of at least 18 dB (Klimley and Myrberg 1979). Therefore, to elicit a robust behavioral response, the sound playback level was 140 dB re 1 µPa re 1 m which yielded a source level of approximately 80 dB above the lowest documented ambient values. At a distance of ∼60 m from the source, the SPL was approximately 20–60 dB above the minimum ambient values. This stimulus immediately achieved maximum intensity upon sound production and provided an impulsive sound that elicited a clear and immediate withdrawal response. In previous literature, shark attraction to an acoustic stimulus was the intended result, and achievement depended on the motivation of the animal. In this study, we deliberately presented sharks with a repulsive acoustic stimulus designed to evoke an immediate and robust response regardless of motivational state.

We quantified the sound propagation in all directions around an underwater speaker to establish the decay rate for various frequencies. The measured SPL values were uniform among different hydrophone recordings at similar distances which confirmed that the sound attenuated evenly around the speaker. The SPL data indicated large losses at low frequencies as the signal propagated. For the 200–400 Hz frequencies, the measured decay fit between the model distributions for cylindrical and spherical spreading whereas for the 400–800 Hz frequencies, the decay was beyond the model for spherical spreading (Fig. 4). At the lowest frequency, 100–200 Hz stimulus, the decay exceeded the model for cylindrical spreading (Fig. 4). SPLs from low frequency sounds do not propagate well through shallow water due to the absorption and reflection from the acoustic boundaries of the sandy substrate and the sea surface (Hawkins and Popper 2018). Most of the acoustic energy could have been lost in the medium due to absorption or transferred into the sediment (angle of intromission; Urick 1975). The sound could also have experienced a Lloyd’s mirror effect, where the calm sea surface conditions could have caused a 180-degree phase shift between the direct and surface-reflected sound waves, nearly canceling each other out (Urick 1975; Gerstein et al. 2005). Low frequency wavelengths are too large to fit in the shallow water which could contribute to a low frequency cut off (Urick 1975). Because the SPL was measured *in situ*, it was possible to infer the SPL at any of the shark orientation locations around the speaker. Ideally, particle motion would have been measured as well, but this was not practical under the field conditions.

For each stimulus band (100–200 Hz, 200–400 Hz, and 400–800 Hz), the peak energy was at a frequency approximately midway between the maximum and minimum frequencies. For the 100–200 Hz stimulus, the peak frequency was 125 Hz, for example. Despite the greatest acoustic energy being found at these intermediate frequencies, we used the lowest frequency of each stimulus (100, 200, and 400 Hz) to calculate the distance that the near field extended from the source. Using this conservative estimate, we calculated the far field to begin at 45 m for the 100 Hz stimulus, 22.5 m for the 200 Hz stimulus, and 11.3 m for the 400 Hz stimulus (Table 1). For all three stimuli, *C. limbatus* responded from a distance of at least 62 m, demonstrating their ability to orient away from a sound that extended well into the far field.

Tank-based hearing trials do not provide a large enough area to test hearing in the acoustic far field. In addition, tank walls will bounce acoustic stimuli resulting in complex wave forms with properties that differ from the source stimulus (Popper and Hawkins 2021). Therefore, the ideal method to test hearing is to conduct experiments under natural conditions in the field (Popper and Hawkins 2021). Only a single study has noted sharks orienting from the acoustic far field, but the sharks’ responses were unable to be recorded or quantified. An abstract (referenced in Nelson 1967) referred to visual observations of sharks orienting to an underwater transducer from 200+ yards, but the full study was not published (Wisby and Nelson 1964). From the abstract, it was determined that the investigators circled in an airplane which provided a sufficiently broad field of view to observe sharks initiating orientations from a much greater distance than could be observed from the boat. No other studies have recorded and quantified shark hearing in the acoustic far field.

The accessibility of unmanned aerial vehicles provides a method to remotely view from above and thus to quantify orientation distance (Christiansen et al. 2016; Butcher et al. 2021). In-air sound recordings have shown that aerial drones emit low frequencies (60–150 Hz depending on the rotor-revolutions), but underwater levels are quantifiable only when the drone is flown at less than 5–10 m above the surface (Christiansen et al. 2016). In this study, the drone was maintained at an altitude of ∼40–50 m where it was undetectable by the sharks and thus could not influence their behavior.

### Response orientation

Prior to the onset of the sound stimulus, sharks swam at a mean trajectory of 63.4° from the speaker, an orientation that was approximately the same among the stimuli (Fig. 7A). This angle was taken from sharks swimming toward or away from the speaker, limiting the trajectory to a 180° plane. Upon sound detection, sharks made a sudden and dramatic turn of 72.7° resulting in a mean trajectory of 136.1° away from the speaker (Movie 1; Fig. 7B). This robust response was consistent for all three test stimuli and provides evidence that sharks can obtain directional information from sound in the far field. The consistent change in direction might represent the maximum angle that the sharks can reorient based on their body flexibility. Sharks with different cross-sectional shapes have different values for second moment of area that might permit some species to bend laterally to a greater extent than others (Kajiura et al. 2003). It would be of interest to test a more flexible species, such as a hammerhead, to determine if it exhibited a greater change in trajectory away from the speaker.

### Response distance

From the drone footage, we measured the distance between the sharks and the speaker when sound was produced. The response distance did not differ between the 100–200 Hz and 200–400 Hz frequency ranges, but sharks did respond to those lower frequencies at distances significantly farther from the sound source, than they did to the 400–800 Hz frequency range. The lack of difference between the low (100–200 Hz) and intermediate (200–400 Hz) frequency ranges might be attributable to a smaller sample size of distant sharks tested with the 100–200 Hz stimulus. If a greater number of distant sharks were sampled for the lower frequency, then the orientation distance might have been statistically greater.

Although the stimuli were constrained to distinct frequency bands (100–200 Hz, 200–400 Hz, and 400–800 Hz), there was still some sound energy produced outside of those bands (Fig. 3). This was particularly apparent at the lower frequencies where stimuli from 100ṭo 200 Hz could bleed over to frequencies below 100 Hz. Because the distance to which the acoustic near field extends is inversely proportional to frequency, sound energy from frequencies below the expected frequency band would produce a near field stimulus at a distance greater than that calculated for the frequency band. As a result, some of the orientations initiated from the presumed far field might have resulted from particle motion extending beyond what was modeled based on the frequency band. Despite this caveat, there was a large number of orientations from higher frequencies (400–800 Hz) that could not be due to bleed over to slightly lower frequencies. Thus, the overall conclusion that the sharks responded to sound in the far field remains valid.

The measured SPLs around the speaker allowed us to determine the SPL at the sharks, whether they responded or not. The lower frequency stimulus required a lower SPL to elicit a response (Fig. 9). This supports previous studies that found that sharks respond best to low frequency stimuli and require a greater stimulus intensity to respond at higher frequencies (Kritzler and Wood 1961; Nelson 1967; Kelly and Nelson 1975; Casper and Mann, 2006, 2009).

### Far field sound detection

We saw responses from sharks in both the near field and the far field. Detecting sound pressure in the far field is thought to be unique to fish that contain a gas-filled swim bladder, which functions as a pressure-to-displacement transducer (Casper 2006; Radford et al. 2012). The gas inside a swim bladder is cyclically compressed and expanded when exposed to oscillating sound pressures, and the sound is thus transformed into particle motion, and reradiated to the inner ear (Yan et al. 2000; Casper 2006; Radford et al. 2012). Elasmobranchs lack this gas-filled organ and therefore cannot rely on this mechanism to detect sound pressure in the far field. They must rely solely on the particle motion component to detect sound in the far field.

In addition to the otolithic end organs of the inner ear, it has been proposed that sharks are also able to detect sound with their macula neglecta (Lowenstein and Roberts 1951). The macula neglecta is highly sensitive to vibrations and gravitational changes and has been documented to be particularly sensitive in sharks (Lowenstein and Roberts 1951). Immediately under the skin on the dorsal surface of the head is an area of loose connective tissue known as the parietal fossa (Lowenstein and Roberts 1951; Tester et al. 1972). The parietal fossa is bounded on the dorsal surface by the skin and on the ventral and lateral surfaces by the cartilaginous neurocranium (Lowenstein and Roberts 1951). Perforating the neurocranium is a bilateral pair of small window-like structures, the fenestra ovali, that consists of tough membranous sheets that form a tympanum-like structure (Lowenstein and Roberts 1951; Tester et al. 1972). The other side of each fenestra ovalis opens directly over the macula neglecta (Lowenstein and Roberts 1951). It has been proposed that sound pressure impinging on the dorsal surface of the head would transmit through the parietal fossa where it would be constrained by the neurocranium and able to displace only the tympanum of the fenestra ovalis (Corwin 1989). The large area of skin overlying the parietal fossa and small window of the fenestra ovalis would result in a mechanical amplification of the sound stimulus. The resulting displacement of the fenestra ovalis would cause the endolymph in the inner ear to flow over the macula neglecta resulting in detection of sound pressure from the far field (Tester et al. 1972; Fay et al. 1974; Corwin 1989).

Although the number of sensory hair cells in the macula neglecta has not been quantified for *C. limbatus*, it is likely to possess a similar morphology to its previously studied congeners, *C. amblyrhynchos, C. falciformis, C. melanopterus*, and *C. brachyurus* (Corwin 1978, 1981; Sauer et al. 2023b). These congeners have been reported to contain a much greater number of sensory hair cells in the macula neglecta compared to other elasmobranchs (Corwin 1978, 1981; Sauer et al. 2023b). A greater number of hair cells is hypothesized to result in greater hearing sensitivity (Casper et al. 2003). The much greater number of macula neglecta hair cells could provide a hypothesis for the blacktip sharks’ ability to orient from distances that were calculated to reach the acoustic far field. The macula neglecta could be the specialized mechanism that allows sharks to detect the low levels of particle motion in the far field, where particle motion is not the dominating force. The results of this study provide behavioral evidence to support the hypothesis that carcharhinids possess specialized hearing capabilities compared to other species. Observations during the course of this study support this assertion. Blacknose sharks (*Carcharhinus acronotus*) were also observed from the drone and the species identification was confirmed by direct underwater observation. During sound playback, blacknose sharks exhibited behavioral responses that mirrored those of *C. limbatus*. In contrast, free-swimming nurse sharks (*Ginglymostoma cirratum*) did not exhibit an overt behavioral response during sound production. However, nurse sharks stationed on the substrate appeared to stir when the lower frequency (100–200 Hz, 200–400 Hz) stimuli were played, although the movement might have been coincidental. The SPLs presented in this study were below the sensitivity threshold for *G. cirratum*, which could account for their lack of response (Casper and Mann 2006).

In addition to the elasmobranch species, we also observed the responses of two species of large teleost fishes, tarpon (*Megalops atlanticus*) and crevalle jacks (*Caranx hippos*). Although the stimuli fell within the hearing sensitivity of most fish (10–2000 Hz; Chapman 1973), there was no apparent response from the teleost fishes during any sound production.

Hearing is an important sensory modality, as it can be stimulated from farther distances compared to other sensory modalities. Sound propagates fast and far in a dense aquatic medium. Because sound informs organisms about their environment, it would be evolutionarily advantageous for predators, such as sharks, to be able to detect and localize sound sources. Therefore, it should not be surprising to discover that sharks are capable of orienting to sounds in the acoustic far field. However, the mechanism remains unclear. There remains a need for additional studies involving the effect of sound on shark behavior, and their ability to detect and respond to sounds of various frequencies. As technology advances, so will the ability to improve investigation efforts.

## Supplementary Material

obag033_Supplemental_File

## Data Availability

All data are available from the corresponding author upon request.

## References

[bib1] Banner A . 1972. Use of sound in predation by young lemon sharks, *Negaprion brevirostris* (Poey). Bull Mar Sci 22(2), 251–83.

[bib1a] Barber VC, Yake KI, Clark VF, Pungur J. 1985. Quantitative analyses of sex and size differences in the macula neglecta and ramus neglectus in the inner ear of the skate, Raja ocellata. Cell Tissue Res 241, 597–605. 10.1007/BF00214581

[bib2] Butcher PA, Colefax AP, Gorkin RA III, Kajiura SM, López NA, Mourier J, Raoult V, Skomal G, Tucker J, Walsh AJ et al. 2021. The drone revolution of shark science: a review. Drones 5(1), 8. 10.3390/drones5010008

[bib3] Casper BM . 2006. The hearing abilities of elasmobranch fishes. USF Tampa Graduate Theses and Dissertations.

[bib4] Casper BM, Lobel PS, Yan HY. 2003. The hearing sensitivity of the little skate, *Raja erinacea*: a comparison of two methods. Environ Biol Fishes 68(4), 371–9. 10.1023/B:EBFI.0000005750.93268.e4

[bib5] Casper BM, Mann DA. 2006. Evoked potential audiograms of the nurse shark (*Ginglymostoma cirratum*) and the yellow stingray (*Urobatis jamaicensis*). Environ Biol Fishes 76, 101–8. 10.1007/s10641-006-9012-9

[bib8] Casper BM, Mann DA. 2009. Field hearing measurements of the Atlantic sharpnose shark *Rhizoprionodon terraenovae*. J Fish Biol 75(10), 2768–76. 10.1111/j.1095-8649.2009.02477.x 20738522

[bib9aa] Castro JI . 2011. The sharks of North America. New York: Oxford University Press.

[bib9] Chapman CJ . 1973. Field studies of hearing in teleost fish. Helgoländer wissenschaftliche Meeresuntersuchungen 24(1), 371–90.

[bib10] Chapuis L, Collin SP. 2022. The auditory system of cartilaginous fishes. Rev Fish Biol Fish 32, 521–54. 10.1007/s11160-022-09698-8

[bib3a] Chapuis L, Yopak KE, Radford CA. 2023. From the morphospace to the soundscape: exploring the diversity and functional morphology of the fish inner ear, with a focus on elasmobranchs. J Acoust Soc Am 154(3), 1526–38. 10.1121/10.0020850 37695297

[bib11] Christiansen F, Rojano-Doñate L, Madsen PT, Bejder L. 2016. Noise levels of multi-rotor unmanned aerial vehicles with implications for potential underwater impacts on marine mammals. Front Mar Sci 3, 277. 10.3389/fmars.2016.00277

[bib12] Corwin JT . 1978. The relation of inner ear structure to the feeding behavior in sharks and rays. Scan Electron Microsc II, 1105–12

[bib14] Corwin JT . 1981. Postembryonic production and aging of inner ear hair cells in sharks. J Comp Neurol 201(4), 541–53. 7287934 10.1002/cne.902010406

[bib15] Corwin JT . 1983. Postembryonic growth of the macula neglecta auditory detector in the ray, *Raja clavata*: continual increases in hair cell number, neural convergence, and physiological sensitivity. J Comp Neurol 217(3), 345–56. 10.1002/cne.902010406 6886057

[bib16] Corwin JT . 1989. Functional anatomy of the auditory system in sharks and rays. J Exp Zool 242, 62–74. 10.1002/jez.1402520408

[bib17] Doan MD, Kajiura SM. 2020. Adult blacktip sharks (*Carcharhinus limbatus*) use shallow water as a refuge from great hammerheads (*Sphyrna mokarran*). J Fish Biol 96, 1530–3. 10.1111/jfb.14342 32274798

[bib18] Evangelista C, Mills M, Siebeck UE, Collin SP. 2010. A comparison of the external morphology of the membranous inner ear in elasmobranchs. J Morphol 271(4), 483–95. 10.1002/jmor.10812 20058296

[bib19] Fay RR, Kendall JI, Popper AN, Tester AL. 1974. Vibration detection by the macula neglecta of sharks. Comp Biochem Physiol A Physiol 47(4), 1235–40. 10.1016/0300-9629(74)90097-8 4156278

[bib20] Gerstein E, Blue JE, Forysthe SE. 2005. The Acoustics of Vessel Collisions with Marine Mammals. Proceedings of OCEANS 2005 MTS/IEEE, Washington, DC, USA, Vol. 2, pp. 1190–7. https://doi: 10.1109/OCEANS.2005.1639917.

[bib21] Hawkins AD, Popper AN. 2018. Directional hearing and sound source localization by fishes. J Acoust Soc Am 144(6), 3329–50. 10.1121/1.5082306 30599653

[bib4a] Higgs DM, Radford CA. 2013. The contribution of the lateral line to 'hearing' in fish. J Exp Biol 216(8), 1489–90. 10.1242/jeb.078816 23264489

[bib22] Kajiura SM, Forni JB, Summers AP. 2003. Maneuvering in juvenile carcharhinid and sphyrnid sharks: the role of the hammerhead shark cephalofoil. Zoology 106(1), 19–28. 10.1078/0944-2006-00086 16351888

[bib23] Kajiura SM, Tellman SL. 2016. Quantification of massive seasonal aggregations of blacktip sharks (*Carcharhinus limbatus*) in Southeast Florida. PLoS One 11(3), e0150911. 10.1371/journal.pone.0150911 27027502 PMC4814085

[bib6a] Kelly JC, Nelson DR. 1975. Hearing thresholds of the horn shark, Heterodontus francisci. J. Acoust. Soc. Am. 58(4), 905–9. 10.1121/1.380742 1194551

[bib25] Klimley AP, Myrberg AA Jr. 1979. Acoustic stimuli underlying withdrawal from a sound source by adult lemon sharks, *Negaprion brevirostris* (Poey). Bull Mar Sci 29(4), 447–58.

[bib26] Kritzler H, Wood L. 1961. Provisional audiogram for the shark, *Carcharhinus leucas*. Science 133(3463), 1480–2. 10.1126/science.133.3463.1480 13754400

[bib28] Lowenstein O, Roberts TDM. 1951. The localization and analysis of the responses to vibration from the isolated elasmobranch labyrinth. A contribution to the problem of the evolution of hearing in vertebrates. J Physiol 114(4), 471–89. 14874224 10.1113/jphysiol.1951.sp004638 PMC1392348

[bib29] Meredith TL, Kajiura SM, Newton KC, Tricas TC, Bedore CN. 2022. Advances in the sensory biology of elasmobranchs. pp. 143–76 In: Carrier JC, Simpfendorfer CA, Heithaus MR, Yopak KE, editors. Biology of sharks and their relatives. 3rd ed. Boca Raton, FL: CRC Press.

[bib31] Moulton JM . 1958. The acoustical behavior of some fishes in the Bimini area. Biol Bull 114(3), 357–74. 10.2307/1538991

[bib32] Myrberg AA . 2001. The acoustical biology of elasmobranchs. In: Tricas TC, Gruber SH, editors. The behavior and sensory biology of elasmobranch fishes: an anthology in memory of Donald Richard Nelson. Developments in environmental biology of fishes. Vol. 20. Dordrecht: Springer. 10.1007/978-94-017-3245-1_4

[bib34] Myrberg AA Jr, Gordon CR, Klimley AP. 1978. Rapid withdrawal from a sound source by open-ocean sharks. J Acoust Soc Am 64(5), 1289–97. 10.1121/1.382114

[bib35] Myrberg AA Jr, Ha SJ, Walewski S, Banbury JC. 1972. Effectiveness of acoustic signals in attracting epipelagic sharks to an underwater sound source. Bull Mar Sci 22(4), 926–49.

[bib36] Nedelec SL, Campbell J, Radford AN, Simpson SD, Merchant ND. 2016. Particle motion: the missing link in underwater acoustic ecology. Methods Ecol Evol 7(7), 836–42. 10.1111/2041-210X.12544

[bib37] Nelson DR . 1967. Hearing thresholds, frequency discrimination, and acoustic orientation in the lemon shark, *Negaprion brevirost*ris (Poey). Bull Mar Sci 17(3), 741–68.

[bib38] Nelson DR, Gruber SH. 1963. Sharks: attraction by low-frequency sounds. Science 142(3594), 975–7. 10.1126/science.142.3594.975 17753802

[bib39] Nelson DR, Johnson RH. 1972. Acoustic attraction of Pacific reef sharks: effect of pulse intermittency and variability. Comp Biochem Physiol A Physiol 42(1), 85–95. 10.1016/0300-9629(72)90370-2 4402726

[bib40] Parker GH . 1910. Influence of the eyes, ears, and other allied sense organs on the movements of the dogfish, Mustelus canis (Mitchill), Bull. Bureau Fish 29: 738, 45–57.

[bib42] Popper AN, Fay RR. 1993. Sound detection and processing by fish: critical review and major research questions (Part 1 of 2). Brain Behav Evol 41(1), 14–25. 10.1159/000316111 8431753

[bib43] Popper AN, Hawkins AD. 2018. The importance of particle motion to fishes and invertebrates. J Acoust Soc Am 143(1), 470–88. 10.1121/1.5021594 29390747

[bib44] Popper AN, Hawkins AD. 2021. Fish hearing and how it is best determined. ICES J Mar Sci 78(7), 2325–36. 10.1093/icesjms/fsab115

[bib45] Popper N, Fay R. 1997. Evolution of the ear and hearing: issues and questions. Brain Behav Evol 50(4), 213–21. 10.1159/000113335 9310196

[bib46] Radford CA, Montgomery JC, Caiger P, Higgs DM. 2012. Pressure and particle motion detection thresholds in fish: a re-examination of salient auditory cues in teleosts. J Exp Biol 215(19), 3429–35. 10.1242/jeb.073320 22693030

[bib8a] Sauer DJ, Radford CA, Mull CG, Yopak KE. 2023a. Quantitative assessment of inner ear variation in elasmobranchs. Sci Rep 13(1), 11939. 10.1038/s41598-023-39151-0 37488259 PMC10366120

[bib9a] Sauer DJ, Yopak KE, Radford CA. 2022a. Ontogeny of the inner ear maculae in school sharks (Galeorhinus galeus). Hear Res 424, 108600. 10.1016/j.heares.2022.108600 36087420

[bib10a] Sauer DJ, Yopak KE, Radford CA. 2022b. Ontogenetic development of inner ear hair cell organization in the New Zealand carpet shark cephaloscyllium isabellum. Frontiers in Ecology and Evolution 10, 1034891. 10.3389/fevo.2022.1034891

[bib11a] Sauer DJ, Yopak KE, Radford CA. 2023b. Interspecific variation in the inner ear maculae of sharks. Integr Org Biol 5(1). 10.1093/iob/obad031 PMC10506894 37732173

[bib47] Tester AL, Kendall JI, Milisen WB. 1972. Morphology of the ear of the shark genus *Carcharhinus*, with particular reference to the macula neglecta. Pac Sci 26, 264–74.

[bib48] Urick RJ . 1975. Principles of underwater sound. 3rd ed. New York: McGraw-Hill.

[bib50] Wisby WJ, Nelson DR. 1964 Airplane observations of acoustic orientation in sharks. In: American Fish Society Conference, Session on fish behavior and sensory biology.

[bib51] Yan HY, Fine ML, Horn NS, Colon WE. 2000. Variability in the role of the gasbladder in fish audition. J Comp Physiol A 186(5), 435–45. 10.1007/s003590050443 10879947

[bib49a] Zar JH . 1999. Biostatistical analysis. Pearson Education India.

